# Coexisting Rheumatoid Arthritis and Spinal Muscular Atrophy: A Case Report

**DOI:** 10.7759/cureus.89779

**Published:** 2025-08-11

**Authors:** Mustafa Alhayali, Shahad Al-Baldawi

**Affiliations:** 1 Internal Medicine/Rheumatology, Ibn Sina University of Medical and Pharmaceutical Sciences, Baghdad, IRQ; 2 Rheumatology and Medical Rehabilitation, Center of Spine and Joint Diseases, Baghdad, IRQ; 3 Rheumatology, Al-Yarmouk Teaching Hospital, Baghdad, IRQ

**Keywords:** anti-interleukin-6, autoimmune, methotrexate, neurodegeneration, rare, rheumatoid arthriitis, spinal muscular atrophy (sma), tofacitinib

## Abstract

Rheumatoid arthritis (RA) and spinal muscular atrophy (SMA) are distinct diseases with vastly different pathophysiologic origins: autoimmune and neurodegenerative, respectively. Their co-occurrence is exceedingly rare and, to our knowledge, previously unreported. We report the case of a 41-year-old female with a seven-month history of inflammatory polyarthritis and a six-year history of undiagnosed progressive proximal muscle weakness. Clinical, serological, and electrophysiological findings confirmed diagnoses of RA and SMA type 4. This unique case underscores the importance of comprehensive evaluation in adults presenting with both joint inflammation and neuromuscular weakness. It also highlights the need for multidisciplinary care and calls for further research into shared mechanisms across neurodegenerative and autoimmune diseases.

## Introduction

Rheumatoid arthritis (RA) is an autoimmune disorder characterized by progressive functional decline, systemic involvement, elevated mortality risk, and significant socioeconomic burden [1]. In 2017, the global age-standardized prevalence of RA was estimated at 246.6 cases per 100,000 individuals, representing a 7.4% increase since 1990 [2]. RA arises from a complex interplay of genetic predisposition, environmental exposures, and immunologic mechanisms [3]. Clinically, it is driven by persistent inflammation of the synovial membrane, leading to joint destruction and pain. Additionally, RA’s systemic inflammatory nature contributes to extra-articular manifestations, particularly cardiovascular disease, a major factor in increased mortality [3,4].

Spinal muscular atrophy (SMA), in contrast, is a genetic neuromuscular disease characterized by progressive degeneration of anterior horn cells in the spinal cord, resulting in muscular weakness and atrophy. More than 95% of cases are caused by a homozygous deletion or mutation in the *SMN1* gene on chromosome 5q13, which encodes the SMN protein in alpha motor neurons of the spinal cord, with autosomal recessive inheritance [5]. In the United States, newborn screening programs estimate the incidence of SMA at approximately 1 in 13,862 live births [6]. SMA is classified into four primary types based on age at onset and clinical severity: Type 1 presents in infancy with profound weakness and early mortality if untreated; Type 2 appears in early childhood, allowing sitting but not walking; Type 3 develops later, with initial ability to walk that may decline over time; and Type 4 emerges in adulthood with milder, slowly progressive symptoms [6,7].

In this report, we describe a unique case of coexisting RA and SMA in a single patient, exploring the diagnostic challenges, potential underlying connections, impact on quality of life, and implications for clinical management. The simultaneous presentation of these distinct conditions, one autoimmune and the other neurodegenerative, raises important clinical and mechanistic questions, including the possibility of shared pathways or a coincidental overlap.

## Case presentation

A 41-year-old female presented with a seven-month history of multiple joint pains involving the hands, elbows, and knees, along with early morning stiffness lasting about two to three hours. She reported gradual swelling of both hands and occasional swelling of the knees, which was nonresponsive to ibuprofen. She denied constitutional symptoms, skin rash, sicca symptoms, or Raynaud’s phenomenon.

She also reported a six-year history of slowly progressive difficulty climbing stairs, standing from a sitting position, and performing overhead activities. She experienced occasional muscle cramps but without sensory or sphincter symptoms. The onset of joint symptoms further affected her quality of life, disturbed her sleep, and impacted her psychological well-being.

Her family history revealed that a paternal cousin had a long-standing history of paralysis, was wheelchair-bound, and ultimately passed away due to complications related to COVID-19. She was born to consanguineous parents (first-degree cousins).

**Figure 1:**
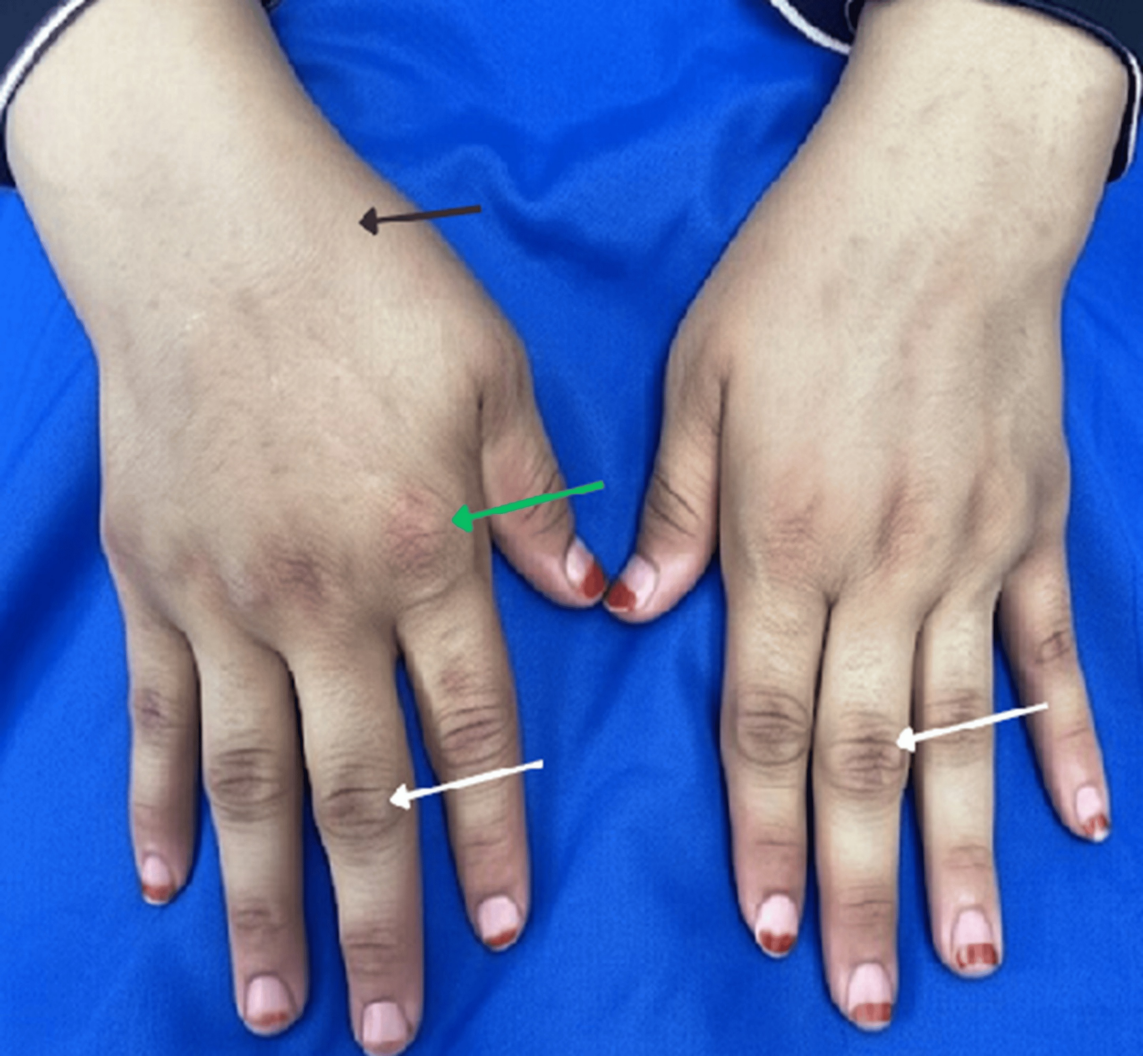
Joint swelling involving the wrists (black arrow) and metacarpophalangeal joints (green arrow), with spindling of the proximal interphalangeal joints (white arrows)

Physical examination revealed joint tenderness and swelling affecting the wrists, metacarpophalangeal joints, and proximal interphalangeal joints (Figure 1), as well as knee effusion. Neurological examination showed reduced power in proximal (grade 3/5) and distal (grade 4/5) muscle groups of the upper and lower limbs, mild weakness in neck flexors, and absent patellar tendon reflexes. Cranial nerve and sensory examinations were intact. The remainder of the physical examination was unremarkable.

Initial blood test results are presented in Table 1.

**Table 1:** Initial blood tests

Parameter	Result	Normal range
White blood cells	5.8 × 10⁹/L	4-10 × 10⁹/L
Hemoglobin	11.1 g/dL	12-16 g/dL
Platelets	699 × 10⁹/L	150-400 × 10⁹/L
Erythrocyte sedimentation rate	48 mm/hour	0-30 mm/hour
C-reactive protein	17 mg/L	<6 mg/L
Rheumatoid factor	124 U/mL	<20 U/mL
Anti-citrullinated peptide antibody	99 U/mL	<30 U/mL
Alanine aminotransferase	37 IU/L	<35 IU/L
Aspartate aminotransferase	14 IU/L	<33 IU/L
Serum creatinine	1.0 mg/dL	0.7-1.4 mg/dL
Anti-nuclear antibody	1:20	<1:40
Creatine kinase	176 μg/L	10-120 μg/L

Electromyography (EMG) and nerve conduction studies (NCS) (Table 2) demonstrated chronic, diffuse anterior horn cell disease of moderate to severe degree, consistent with SMA type 4.

**Table 2:** NCS and EMG of the upper and lower limbs showing a neurogenic pattern consistent with anterior horn cell disease APB: abductor pollicis brevis; DML: distal motor latency; EDB: extensor digitorum brevis; EMG: electromyogram; FDI: first dorsal interosseous; MNCV: motor nerve conduction velocity; MUAPs: motor unit action potentials; NCS: nerve conduction study; SAP: sensory action potential; SNCV: sensory nerve conduction velocity; TA: tibialis anterior

Test	Nerve/muscle	Finding	Interpretation/notes
Sensory NCS	Rt. Median	Latency 4.2 ms	Prolonged latency
		Amp 12.3 µV	Reduced amplitude
		SNCV 35.5 m/s	Reduced velocity
	Lt. Median	Absent SAP	No sensory response
	Rt. Ulnar	Latency 2.3 ms	Normal parameters
		Amp 25.6 µV	Normal parameters
		SNCV 56.6 m/s	Normal parameters
	Lt. Sural	Latency 2.4 ms	Normal parameters
		Amp 15.5 µV	Normal parameters
		SNCV 44.3 m/s	Normal parameters
Motor NCS	Rt. Median (APB)	DML 5.6 ms	Prolonged distal latency
		MNCV 55.3 m/s	-
		F-wave 27.2 ms	-
	Lt. Median (APB)	DML 7.4 ms	Prolonged latency
		MNCV 53.3 m/s	-
		F-wave 27.7 ms	-
	Rt. Ulnar (FDI)	DML 3.7 ms	Reduced conduction velocity
		MNCV 56.3 m/s	-
		F-wave 28.7 ms	-
	Rt./Lt. Common peroneal (EDB)	No response	Bilateral absence of response
	Rt. Tibial (TA)	DML 5.2 ms	Reduced CMAP amplitude
	Lt. Tibial (TA)	DML 5.4 ms	Reduced CMAP amplitude
Needle EMG	Upper trapezius	Examined	No abnormal findings mentioned
	Deltoid (Rt.)	Duration 22.8 ms (↑)	Long duration motor unit potentials
	Biceps (Rt.)	Duration 24.9 ms (↑)	Long duration motor unit potentials
	Vastus medialis (Rt.)	Duration 25.2 ms (↑)	Long duration motor unit potentials
	Vastus medialis (Lt.)	Duration 26.2 ms (↑)	Long duration motor unit potentials
	Tibialis anterior (Rt.)	Duration 27.2 ms (↑)	Long duration motor unit potentials
	Tibialis anterior (Lt.)	Duration 27.3 ms (↑)	Long duration motor unit potentials
	All muscles sampled	+ve Sharp waves	Neurogenic pattern
		Fibrillation	Denervation signs suggest anterior horn cell disease
		Polyphasia (40-30%)	-
		Reduced recruitment	-
		Unstable MUAPs	-

**Figure 2:**
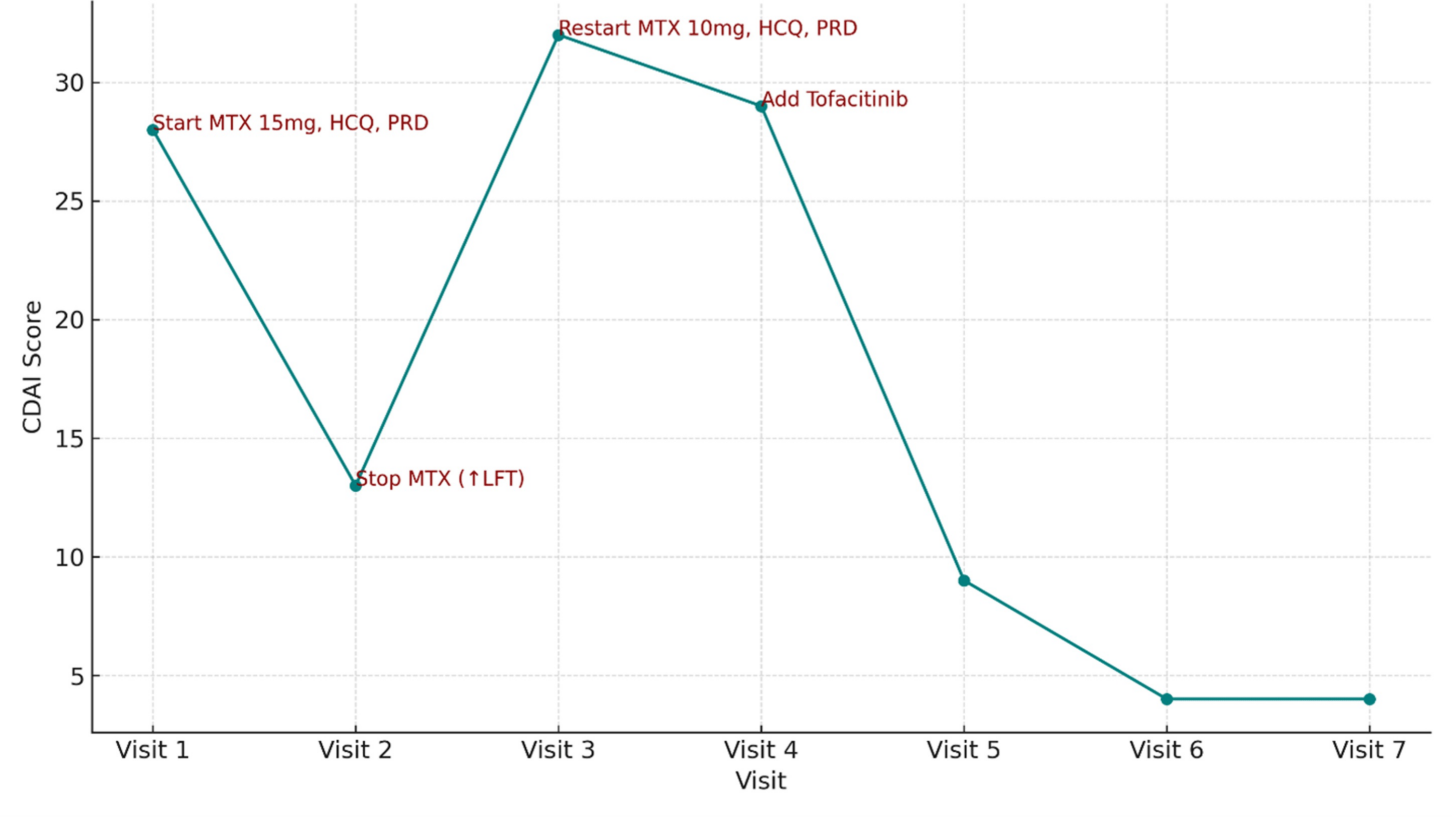
CDAI progression over time with treatment modifications CDAI: Clinical Disease Activity Index

Concurrently, the patient fulfilled the ACR/EULAR 2010 classification criteria for RA, with a Clinical Disease Activity Index (CDAI) score of 28, indicating high disease activity. Figure 2 illustrates the timeline of her CDAI scores.

Initial treatment included oral methotrexate (15 mg/week), hydroxychloroquine (200 mg/day) adjusted to body weight, and prednisolone (10 mg/day). After four weeks of therapy, the patient experienced symptomatic improvement. However, liver function tests revealed elevated liver enzymes, necessitating temporary discontinuation of methotrexate for two weeks. Once liver enzymes normalized, methotrexate was reintroduced at a reduced dose of 10 mg/week.

Despite these adjustments, the patient’s disease activity persisted. Therefore, tofacitinib (5 mg twice daily) was initiated, and hydroxychloroquine was continued alongside reduced-dose methotrexate, resulting in complete clinical remission. At her most recent follow-up visit, the patient maintained sustained remission, which permitted a gradual tapering of prednisolone.

## Discussion

SMA type 4 is a rare, adult-onset form characterized by symmetrical proximal muscle weakness and atrophy, typically beginning after age 30 and progressing slowly [8]. Unlike the more severe SMA types 1 and 2, which are often fatal due to respiratory failure, type 4 generally allows for a near-normal life span [9]. Pathologically, SMA results from mutations in the *SMN1 *gene, leading to reduced levels of SMN protein, which plays an essential role in RNA metabolism and is particularly abundant in motor neurons [10]. Affected individuals show a marked reduction in nuclear structures known as GEMS (Gemini of coiled bodies) [11].

While SMA is classically considered a monogenic neurodegenerative disorder, recent studies suggest an immune-mediated component, particularly in severe cases. Elevated cerebrospinal fluid levels of pro-inflammatory cytokines, including IL-6, interferon gamma, tumor necrosis factor alpha (TNFα), and others, have been reported in SMA type 1, highlighting a potential role for neuroinflammation in disease progression [12]. IL-6 and TNFα, in particular, are also central to the pathogenesis of RA, where they promote synovial inflammation, angiogenesis, and joint destruction [13]. The shared elevation of these pro-inflammatory molecules in both RA and SMA raises the possibility of overlapping inflammatory mechanisms. In RA, IL-6 inhibition with biologics such as tocilizumab is well established [13], and TNFα inhibitors are widely used in the treatment of moderate to severe disease, whereas in SMA, this inflammatory axis remains an emerging area of interest.

Our patient’s rare co-presentation of RA and SMA type 4 invites speculation about a common genetic or immunologic susceptibility. For example, polymorphisms in genes such as IL-6 may influence both neurodegeneration and autoimmunity. It is unclear whether RA developed independently or was facilitated by SMA-related immune dysregulation, but the association appears theoretically possible.

In addition to SMA, the presence of polyarthritis and muscle weakness can be attributed to other pathologies, such as idiopathic inflammatory myopathies, systemic lupus erythematosus (SLE), drug-induced myopathy, or malignancy. However, the patient’s history, clinical examination, and initial investigations effectively excluded drug exposure, SLE, and malignancy. Furthermore, the strong family history and the finding of anterior horn cell disease on EMG/NCS pointed toward SMA.

While RA and SMA are individually well documented, their co-occurrence is exceedingly rare and has not been previously reported. A potential explanation lies in the significantly shortened life span associated with severe SMA, which may prevent affected individuals from reaching the age at which RA commonly develops. Our patient’s diagnosis of SMA was based on clinical presentation, family history, and neurophysiological studies; genetic testing was not performed due to lack of facilities.

Currently approved SMA treatments, including nusinersen, risdiplam, and gene therapy, focus on increasing SMN protein levels but do not directly address neuroinflammation. If inflammation contributes to disease severity in SMA, as suggested by elevated IL-6, future therapies could consider immunomodulation as an adjunctive strategy. Conversely, management of RA in patients with neuromuscular comorbidities requires careful selection and monitoring of immunomodulatory therapies, considering both systemic side effects and potential implications for neuromuscular function. For example, TNF inhibitors are potentially associated with demyelination [14], which is an important concern in treating RA patients, especially those at risk. The coexistence of muscle weakness and arthritis in this patient represents an additional challenge, and a multidisciplinary approach is necessary to reduce musculoskeletal symptom severity and their impact on daily activities. Furthermore, SMA patients may develop scoliosis and/or kyphosis, hip dislocation, and joint contractures [15].

To our knowledge, this is the first reported case of coexisting SMA and RA. While these conditions differ in origin, one genetic and neurodegenerative, the other autoimmune, their intersection in a single patient highlights both diagnostic complexity and cumulative functional burden. This case underscores the importance of thorough evaluation when neuromuscular symptoms coexist with systemic inflammation and reminds us that rare combinations can have profound real-life implications. Notably, motor neuron disease, another neurodegenerative condition, has also been reported concurrently in patients with RA [16,17], further supporting a possible correlation between neurodegeneration and autoimmunity.

## Conclusions

This case highlights the unusual co-occurrence of RA and SMA type 4, conditions with distinct underlying mechanisms. It underscores the need to maintain a broad differential diagnosis in adults presenting with both joint inflammation and long-standing muscle weakness. Whether this overlap is coincidental or reflects shared inflammatory pathways remains uncertain; however, it raises important questions about immune involvement in neurodegenerative disorders. Above all, it emphasizes the importance of holistic, multidisciplinary care in managing complex patients. Future research directions could include genetic studies to identify potential shared susceptibility loci between RA and SMA, as well as investigations of inflammatory markers in larger SMA cohorts.
